# Causal analysis of dietary preferences and the risk of endometriosis using large-scale population data

**DOI:** 10.1038/s41598-025-86707-3

**Published:** 2025-01-21

**Authors:** Xin Cheng, Dan Ma, Xiuhong Wang, Meiling Li, Jinpeng Jiang

**Affiliations:** Department of Gynaecology, Xianning Central Hospital, The First Affiliated Hospital of Hubei University of Science and Technology, 228 Jingui Road, Xianan District, Xianning, 437100 China

**Keywords:** Endometriosis, Dietary, Mendelian randomization, Asparagus, Coffee, Orange juice, Nutrition, Lifestyle modification, Data mining

## Abstract

Dietary factors have recently been recognized as potentially influential in the pathogenesis of endometriosis (EM), yet studies on the causal relationship between dietary preferences and EM are limited. The present study aimed to explore the causal relationships between 187 dietary preferences and EM using Mendelian randomization (MR) methods. This study utilised genome-wide association study data from over 500,000 European participants for dietary preferences and 64,658 EM patients from Finland. Dietary preferences with potential causal relationships to EM were identified using two-sample MR methods. P-values from the inverse variance weighted (IVW) analysis were corrected using the false discovery rate (FDR) method to ensure accuracy. Additionally, heterogeneity analysis, pleiotropy assessment, leave-one-out analysis, and reverse MR analysis were conducted to further validate and solidify the findings of the study. After FDR correction, IVW analysis revealed that asparagus preference was significantly protective against EM, including in American Society for Reproductive Medicine (ASRM) stages 1–2 and 3–4. Conversely, preferences for different types of coffee and orange juice were associated with an increased likelihood of EM across these stages. Subgroup analysis indicated that asparagus preference had a protective effect against deep EM, ovarian EM, pelvic peritoneal EM, and rectovaginal septum and vaginal EM. In contrast, coffee preference increased the risk of pelvic peritoneal EM, fallopian tube EM, and unspecified EM, whereas orange juice preference increased the risk of deep EM, rectovaginal septum and vaginal EM, pelvic peritoneal EM, and unspecified EM. Reverse MR analysis did not identify causal relationships between EM and the specific dietary preferences that were analysed in this study. These findings suggest that asparagus preference significantly reduces the risk of developing EM, whereas preferences for orange juice and different types of coffee may increase the risk, offering new insights into EM management through dietary modifications.

**Supplementary Information:**

The online version contains supplementary material available at 10.1038/s41598-025-86707-3.

## Introduction

Endometriosis (EM) is a prevalent gynaecological disorder affecting approximately 10–15% of women of reproductive age worldwide, equating to approximately 190 million women globally^1–3^. The primary clinical symptoms of EM include chronic pelvic pain, dysmenorrhea, dyspareunia, dyschezia, infertility, and irregular menstrual bleeding^4–6^. These symptoms severely impact patients’ quality of life and mental health^7^. The exact aetiology of EM remains unclear, but it is believed to involve a complex interplay of genetic, immune, endocrine, and environmental factors^8^. The unknown aetiology of EM poses significant challenges for its diagnosis and treatment. Current treatments include pharmacological interventions with limited efficacy and surgical procedures, where traditional white-light surgery may miss small or hidden lesions, contributing to high recurrence rates^9,10^. Studies have shown that the five-year recurrence after surgical treatment can reach 40–50%, whereas the recurrence rate after pharmacological treatment is as high as 50–70%^11–13^. Therefore, further research is urgently needed to explore new prevention and treatment strategies to improve the prognosis and quality of life of EM patients.

In recent years, dietary factors have garnered significant attention as modifiable lifestyle elements potentially influencing the pathogenesis of EM^14,15^. Numerous observational studies suggest that specific dietary patterns may be associated with the risk and severity of EM symptoms. For example, research indicates that a high intake of fruits, vegetables, whole grains, and omega-3 fatty acids may reduce the risk of developing EM, whereas high consumption of red meat, caffeine, alcohol, and high-fat foods may increase the risk^16–18^. A prospective study involving 35 women with EM revealed that following a Mediterranean diet for three months significantly alleviated their pain symptoms^19^. Another case‒control study involving 78 EM and 78 non-EM women revealed that dietary intake of calcium, potassium, and vitamins B12, B2, B6, and C could effectively reduce the risk of EM^20^. Furthermore, dietary factors are crucial for preventing and managing various gynaecological conditions. Specifically, a randomised controlled trial (RCT) demonstrated that dietary interventions could alleviate pain in female university students with primary dysmenorrhea^21^. Additionally, fruit intake may reduce the risk of uterine fibroids, and a ketogenic diet, by reducing carbohydrate intake, can effectively improve symptoms of polycystic ovary syndrome (PCOS)^22,23^. These findings underscore the importance of diet in the prevention and management of gynaecological diseases. However, studies exploring the causal relationship between diet and EM remain limited, with a lack of large-scale, high-quality research to clarify the specific impact of diet on EM progression. Therefore, further research into the mechanisms by which dietary factors influence EM could provide essential scientific evidence for developing new interventions and guiding patients in formulating personalised dietary plans.

Given the impracticality of conducting large-scale RCTs to study the impact of dietary factors on EM, Mendelian randomization (MR) offers an innovative research approach. MR utilises genetic variants as instrumental variables to assess causal relationships between exposures and outcomes, effectively reducing the influence of confounding factors and reverse causation and thereby enhancing the reliability of the findings^24^. Therefore, this study employs MR methods to systematically investigate the causal relationships between various dietary preferences and the risk of developing EM, aiming to fill this research gap and provide scientific evidence for the clinical management of and dietary guidance for patients.

## Materials and methods

### Study objective and design

**Fig. 1:**
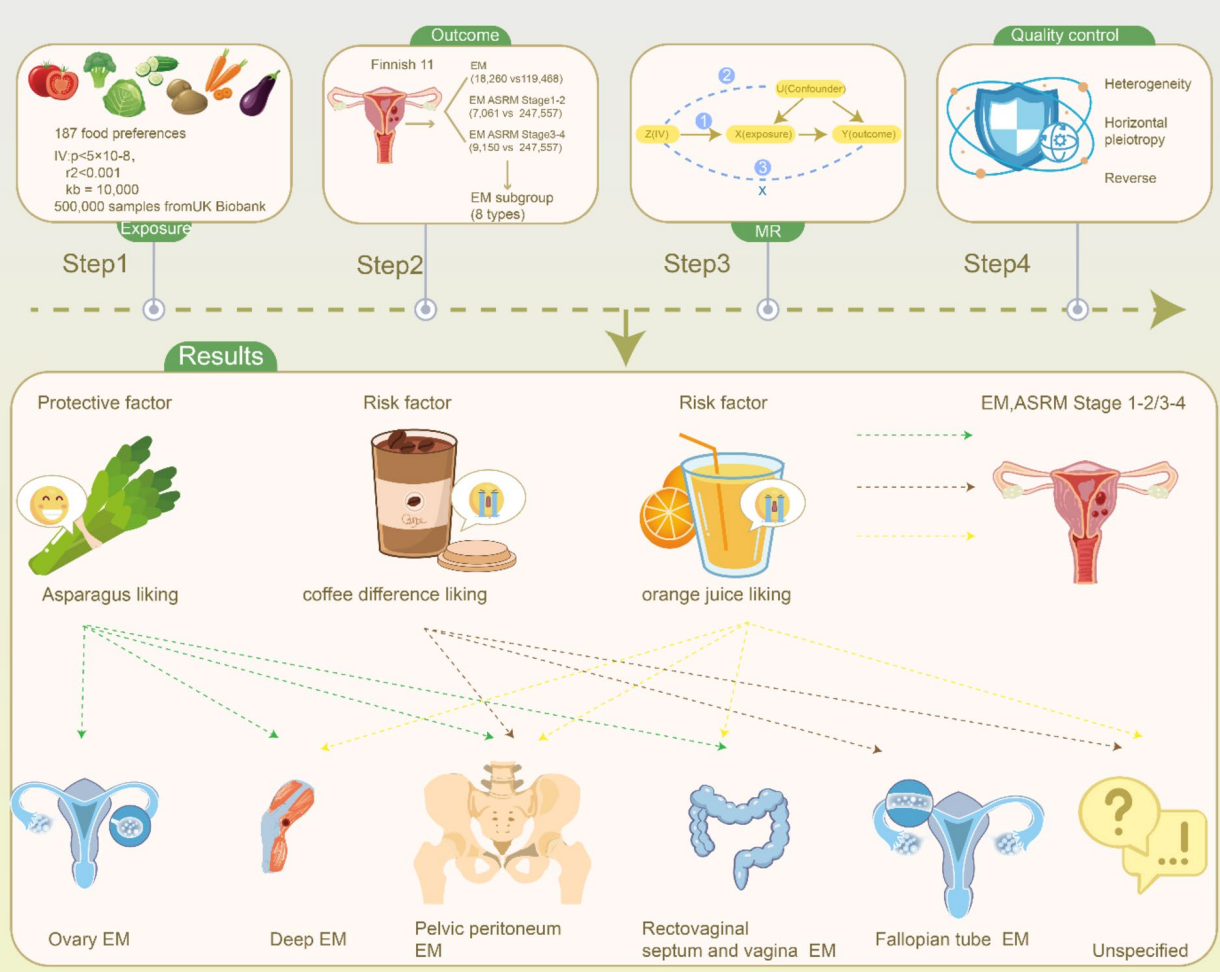
Flowchart of the present study. *MR* Mendelian randomization, *EM* endometriosis, *ASRM* American Society for Reproductive Medicine.

The aim of this study was to explore the potential causal relationships between different dietary preferences (as exposure factors) and EM using bidirectional MR analysis. This study adhered to the standards outlined in the Strengthening the Reporting of Observational Studies in Epidemiology (STROBE) checklist (Table S1)^25^. Compared with retrospective studies, MR analysis provides more reliable results by reducing various bias factors. However, the accuracy of MR analysis depends on the complete implementation of three fundamental principles: first, the instrumental variables (IVs) must have a strong correlation with the exposure factors; second, there should be no significant association between the IVs and confounding factors; and third, the IVs should not directly affect the outcome factors^26–28^. To ensure the implementation of these principles, we employed various methodologies in the study design. We validated the effectiveness of the IVs through *P*-value filtering, F-statistic calculations, and linkage disequilibrium (LD) filtering. Additionally, techniques such as Steiger filtering, MR-PRESSO filtering, and the removal of single nucleotide polymorphisms (SNPs) associated with confounding factors were applied to minimise horizontal pleiotropy. Calculations including Egger intercept, leave-one-out, and various sensitivity analysis methods further ensured the stability of the results. Finally, we addressed reverse confounding bias through reverse MR analysis and Steiger testing, as illustrated in Fig. 1.

### Data sources

This study utilised data from the UK Biobank project 19,655 for dietary preferences^29^ and the Finnish database version R11 (ICD-10: N14) for EM data (available at https://r11.finngen.fi/). The dietary preference dataset was derived from an online questionnaire completed by over 500,000 UK Biobank participants of European ancestry. This questionnaire is an expanded version of a previous one, consisting of 152 items, 139 of which are related to the food and beverages used in this study. Preferences for coffee and tea were measured both with and without sugar, generating two additional variables: the maximum preference score (coffee max and tea max) and the difference between sweetened and unsweetened scores, indicating polarization in preference. These adjustments resulted in 144 items. Additionally, food preferences with shared characteristics, such as desserts, meat, and savory items, were grouped into categories such as “F-Highly Palatable”, adding 43 items. In total, 187 dietary preference traits were analysed^30,31^. The detailed food preference data IDs and corresponding phenotypic information can be found in Table S2. The EM data were obtained from the Finnish database and included information on various stages and anatomical locations of EM (Table S3). The summary statistics used in this study were derived from publicly available databases. All related genome-wide association study (GWAS) summary statistics were approved by relevant ethics committees, and informed consent was obtained from participants in the original studies. Thus, no further ethical approval was required for this study. All methods were carried out in accordance with relevant guidelines and regulations.

### Instrumental variable selection

The conditions for the significant association of instrumental genetic variants included: *p* < 5 × 10^− 8^, *r*^2^ < 0.001, a genetic distance of 10,000 kb, and all F-test values > 10^32–34^. All phenotypes associated with the instrumental variables were examined, and SNPs related to the outcome factors (*p* < 5 × 10^− 8^) were excluded to ensure validity across multiple analyses. Additionally, to minimise the influence of confounding factors on causal effects, we utilised the LDlink webtool (https://ldlink.nih.gov/?tab=ldtrait) to retrieve relevant traits of strongly correlated SNPs and excluded these SNPs from the analysis, as detailed in Table S4. In the forwards univariate MR analysis, the exposure factors consisted of 187 dietary preference data points, with EM as the outcome. For the reverse MR analysis, EM was treated as the exposure factor, while dietary preference data were considered the outcomes. The parameters were set the same as those in the forwards univariate MR analysis.

### Mendelian randomization methods

To increase the reliability and accuracy of our study results, we employed four different MR methods to assess causal relationships. The primary method used was the inverse-variance weighted (IVW) approach, which calculates causal effects by taking the weighted average of wald ratios across all SNPs. IVW provides the most efficient estimates under the assumption that all instrumental variables (SNPs) are valid, integrating multiple data sources and improving statistical power through variance balancing. However, IVW is sensitive to pleiotropy, which can introduce bias if present^35,36^. To address the potential issue of pleiotropy, we supplemented our analysis with the MR-Egger regression method. MR-Egger can detect and correct for pleiotropic effects, offering reliable causal estimates even when all SNPs exhibit pleiotropy. Its distinctive feature is the ability to adjust for bias by examining the regression intercept for evidence of pleiotropy^37^. In addition, we utilised the weighted median method, a robust statistical technique that is particularly effective when some SNPs may be invalid. This method calculates the causal effect by weighting the median of SNP effects, thereby minimising the influence of outlier SNPs. It allows up to 50% of the instrumental variables to be invalid while still providing accurate causal effect estimates, demonstrating strong robustness in the presence of pleiotropy or data noise^38^. Finally, we employed the weighted mode method, which estimates causal effects by identifying the most common mode of SNP effects. This method is particularly useful in situations where many instrumental variables are invalid or pleiotropic. It excels in accurately identifying true causal directions even when a majority of SNPs are not valid^39^. In summary, when all four methods have consistent effect directions, we consider the MR results to be robust. By integrating these methods, we can comprehensively assess the existence of causal effects and mitigate the risk of bias introduced by relying on a single method, thereby reducing the likelihood of erroneous conclusions.

### Quality control

To ensure the robustness and reliability of the MR analysis, the results with fewer than three available SNPs were excluded from the analysis. Additionally, we conducted heterogeneity and horizontal pleiotropy tests. A *P*-value less than 0.05 in Cochran’s Q test indicates the presence of heterogeneity^36^. MR Egger regression was utilised to assess horizontal pleiotropy, with a significant intercept *P*-value (*p* < 0.05) suggesting pleiotropy^37^. Furthermore, we employed a “leave-one-out” approach, sequentially removing each SNP and recalculating the meta-effects to determine if the results remained consistent. If the overall effect size remained stable after each SNP was removed, the analysis was considered robust^40^.

### Statistical analysis

All analyses in this study were conducted using R software (version 4.3.1). We employed several R packages, including ‘TwoSampleMR’ (version 0.6.0), ‘MendelianRandomization’ (version 0.8.0), and ‘MRPRESSO’ (version 1.0) to perform the MR analyses. In the initial phase, the results with P values less than 0.05 obtained via the IVW method were considered to have potential causal relationships. To ensure the accuracy of these causal inferences, we applied multiple comparison corrections using the false discovery rate (FDR) method. Postcorrection, only those results with P values still less than 0.05 were regarded as truly indicative of causal relationships. The final outcomes are reported as odds ratios (ORs) with corresponding 95% confidence intervals (CIs).

## Results

### Genetic instrumental variables

A total of 187 dietary preferences were initially considered as exposure variables, with 12 types of EM serving as outcomes. Instrumental variables were selected on the basis of *p* < 5 × 10^− 8^, *r*^2^ < 0.001, and a genetic distance of 10,000 kb. Harmonization and Steiger filtering were applied to exclude weak instrumental variables and outliers. Additionally, confounding SNPs associated with potential confounders were removed, as detailed in Table S4. Following these exclusions, nine dietary preferences were excluded because of insufficient instrumental SNP coverage, leaving 178 dietary phenotypes for further analysis. In the final analysis, these 178 dietary phenotypes corresponded to 18,499 unique SNP‒outcome combinations. This total reflects the application of individual SNPs across multiple dietary preferences and EM outcomes, where a single SNP linked to a dietary preference could be tested across several EM outcomes. The final set of instrumental SNPs used in the analysis is provided in Table S5.

### Core MR analysis results for the EM

**Fig. 2:**
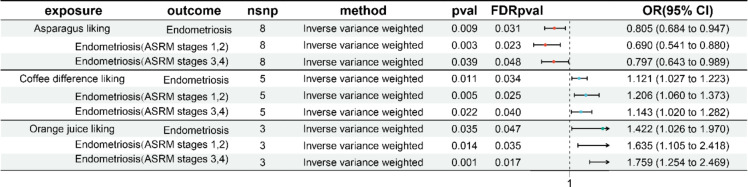
Forest plot of the MR study between dietary link and EM. *MR* Mendelian randomization, *EM* endometriosis.

The results of the MR analysis of EM revealed that, after FDR correction, three dietary preferences exhibited a protective causal relationship with EM: asparagus liking (*p* = 0.009, FDR *p* = 0.031, *OR* = 0.805, *95% CI*: 0.684–0.947), diet fizzy drink liking (*p* = 0.010, FDR *p* = 0.033, *OR* = 0.727, *95% CI*: 0.570–0.926), and mackerel liking (*p* = 0.022, FDR *p* = 0.040, *OR* = 0.891, *95% CI*: 0.807–0.983). Additionally, three dietary preferences showed a risk causal relationship with EM: orange juice liking (*p* = 0.035, FDR *p* = 0.047, *OR* = 1.422, *95% CI*: 1.026–1.970), coffee difference liking (*p* = 0.011, FDR *p* = 0.034, *OR* = 1.121, *95% CI*: 1.027–1.223), and whiskey liking (*p* = 0.012, FDR *p* = 0.034, *OR* = 1.321, *95% CI*: 1.063–1.642). Notably, only asparagus liking, coffee difference liking, and orange juice liking demonstrated causal relationships with EM in ASRM stages 1–2 and 3–4, which is consistent with the overall EM analysis results (protective or risk trends), as shown in Fig. 2. In conclusion, the analysis revealed that the dietary preferences for asparagus, coffee difference, and orange juice have consistent impacts on different stages of EM.

### Subgroup analysis

To further investigate the impact of asparagus liking, coffee difference liking, and orange juice liking on different sites of EM, we conducted a subgroup analysis involving nine specific locations: deep EM, EM of the fallopian tube, EM of the intestine, unspecified/other EM, EM of the ovary, EM of the pelvic peritoneum, EM of the rectovaginal septum and vagina, skin scar EM, and adenomyosis (EM of the uterus). The results indicated that asparagus liking had a protective effect on deep EM (*p =* 0.020, FDR *p =* 0.040, *OR* = 0.664, 95% *CI*: 0.470–0.939), EM of the ovary (*p =* 0.042, FDR *p =* 0.048, *OR* = 0.773, 95% *CI*: 0.603–0.991), EM of the pelvic peritoneum (*p =* 0.002, FDR *p =* 0.020, *OR* = 0.646, 95% *CI*: 0.492–0.849), and EM of the rectovaginal septum and vagina (*p =* 0.012, FDR *p =* 0.034, *OR* = 0.613, 95% *CI*: 0.419–0.897). Coffee differences liking significantly affected EM of the fallopian tube (*p =* 0.005, FDR *p =* 0.025, *OR* = 2.606, 95% *CI*: 1.342–5.061), unspecified/other EM (*p =* 0.007, FDR *p =* 0.029, *OR* = 1.327, 95% *CI*: 1.080–1.631), and EM of the pelvic peritoneum (*p =* 0.007, FDR *p =* 0.029, *OR* = 1.204, 95% *CI*: 1.053–1.377). Similarly, orange juice liking had significant effects on deep EM (*p =* 0.035, FDR *p =* 0.047, *OR* = 1.422, 95% CI: 1.026–1.970), unspecified/other EM (*p =* 0.017, FDR *p =* 0.039, *OR* = 1.932, 95% *CI*: 1.127–3.311), EM of the pelvic peritoneum (*p =* 0.035, FDR *p =* 0.047, *OR* = 1.788, 95% *CI*: 1.041–3.072), and EM of the rectovaginal septum and vagina (*p =* 0.0002, FDR *p =* 0.007, *OR* = 3.077, 95% *CI*: 1.691–5.598. The results from IVW and other MR methods are detailed in Table S6. Additionally, MR analysis findings for 178 dietary phenotypes and all EM types, including FDR-adjusted *P-*values for variables with *p* < 0.05, are also provided in Tables S7-S8. These findings suggest that asparagus liking, different coffee liking, and orange juice liking have consistent effects on different sites of EM, highlighting their significant impacts on various EM locations.

### Quality control

**Fig. 3:**
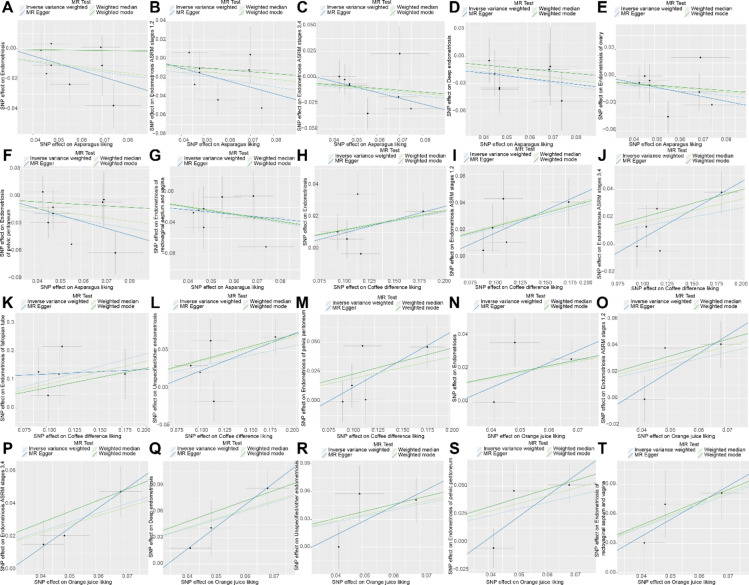
Scatter plot showing the effects of three dietary preferences on EM identified by IVW.

**Fig. 4:**
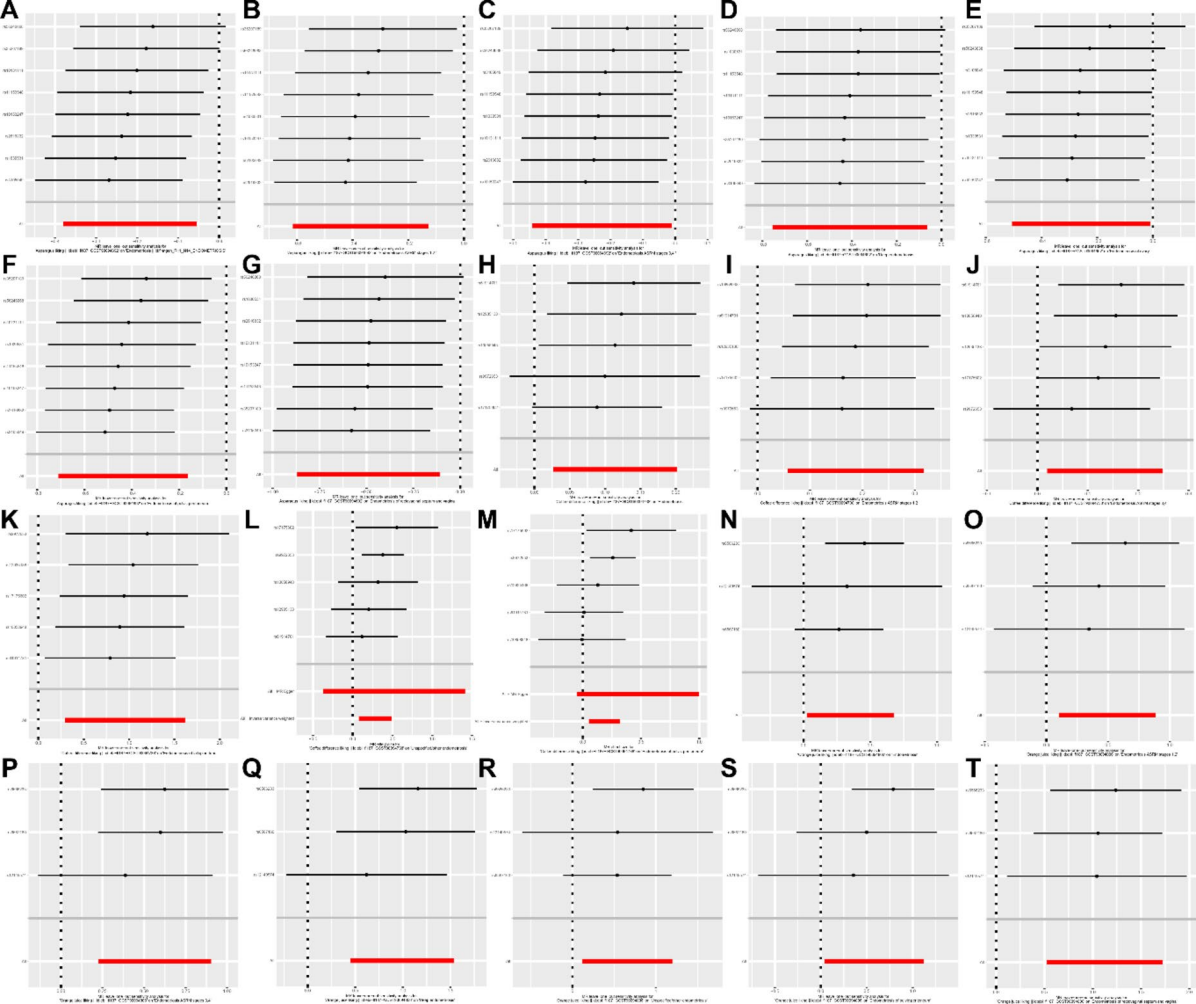
Leave-one-out plots for the causal associations between three dietary preferences and EM.

To ensure the reliability of our analysis results, we conducted heterogeneity, sensitivity, and horizontal pleiotropy analyses. The scatter plot (Fig. 3) demonstrated consistency in the analysis results. As shown in Table 1, the results of the Cochran Q test indicated no heterogeneity, and the pleiotropy test revealed no horizontal pleiotropy among the SNPs (*p* > 0.05) (Table 2). Additionally, we employed the leave-one-out method to assess the impact of potential outliers on the causal effects of asparagus liking, coffee difference liking, and orange juice liking on EM. Removing any individual SNP did not significantly alter the results (Fig. 4), supporting the robustness and reliability of our findings.

**Table 1:** Cochran Q test results.

Exposure	Outcome	Method	Q	df	*P*
Asparagus liking	Endometriosis	MR Egger	6.143337	6	0.407327
Asparagus liking	Endometriosis	Inverse variance weighted	6.486092	7	0.484269
Asparagus liking	Endometriosis ASRM stages 1,2	MR Egger	6.375233	6	0.382494
Asparagus liking	Endometriosis ASRM stages 1,2	Inverse variance weighted	6.616795	7	0.469838
Asparagus liking	Endometriosis ASRM stages 3,4	MR Egger	5.252477	6	0.511863
Asparagus liking	Endometriosis ASRM stages 3,4	Inverse variance weighted	5.622362	7	0.584469
Asparagus liking	Deep endometriosis	MR Egger	1.765227	6	0.939975
Asparagus liking	Deep endometriosis	Inverse variance weighted	1.779437	7	0.971034
Asparagus liking	Endometriosis of ovary	MR Egger	5.322685	6	0.503141
Asparagus liking	Endometriosis of ovary	Inverse variance weighted	5.475892	7	0.602093
Asparagus liking	Endometriosis of pelvic peritoneum	MR Egger	8.033386	6	0.235668
Asparagus liking	Endometriosis of pelvic peritoneum	Inverse variance weighted	8.238085	7	0.312069
Asparagus liking	Endometriosis of rectovaginal septum and vagina	MR Egger	2.941815	6	0.816113
Asparagus liking	Endometriosis of rectovaginal septum and vagina	Inverse variance weighted	2.979808	7	0.886864
Coffee difference liking	Endometriosis	MR Egger	3.995606	3	0.261939
Coffee difference liking	Endometriosis	Inverse variance weighted	4.090773	4	0.39386
Coffee difference liking	Endometriosis ASRM stages 1,2	MR Egger	1.778928	3	0.619531
Coffee difference liking	Endometriosis ASRM stages 1,2	Inverse variance weighted	2.13639	4	0.71069
Coffee difference liking	Endometriosis ASRM stages 3,4	MR Egger	1.824372	3	0.609646
Coffee difference liking	Endometriosis ASRM stages 3,4	Inverse variance weighted	3.374967	4	0.497143
Coffee difference liking	Endometriosis of fallopian tube	MR Egger	1.615405	3	0.655902
Coffee difference liking	Endometriosis of fallopian tube	Inverse variance weighted	1.998499	4	0.736035
Coffee difference liking	Unspecified/other endometriosis	MR Egger	4.823481	3	0.185189
Coffee difference liking	Unspecified/other endometriosis	Inverse variance weighted	5.290327	4	0.258784
Coffee difference liking	Endometriosis of pelvic peritoneum	MR Egger	2.784498	3	0.426058
Coffee difference liking	Endometriosis of pelvic peritoneum	Inverse variance weighted	4.028381	4	0.402179
Orange juice liking	Endometriosis	MR Egger	2.787409	1	0.095008
Orange juice liking	Endometriosis	Inverse variance weighted	3.240732	2	0.197826
Orange juice liking	Endometriosis ASRM stages 1,2	MR Egger	1.134491	1	0.286819
Orange juice liking	Endometriosis ASRM stages 1,2	Inverse variance weighted	2.084512	2	0.352658
Orange juice liking	Endometriosis ASRM stages 3,4	MR Egger	0.02055	1	0.886013
Orange juice liking	Endometriosis ASRM stages 3,4	Inverse variance weighted	0.759618	2	0.683992
Orange juice liking	Deep endometriosis	MR Egger	0.016859	1	0.896692
Orange juice liking	Deep endometriosis	Inverse variance weighted	1.529048	2	0.465555
Orange juice liking	Unspecified/other endometriosis	MR Egger	1.505134	1	0.219883
Orange juice liking	Unspecified/other endometriosis	Inverse variance weighted	2.06716	2	0.355731
Orange juice liking	Endometriosis of pelvic peritoneum	MR Egger	1.825899	1	0.176613
Orange juice liking	Endometriosis of pelvic peritoneum	Inverse variance weighted	3.741166	2	0.154034
Orange juice liking	Endometriosis of rectovaginal septum and vagina	MR Egger	0.401303	1	0.526417
Orange juice liking	Endometriosis of rectovaginal septum and vagina	Inverse variance weighted	0.578378	2	0.748871

**Table 2:** Egger intercept test results.

Exposure	Outcome	Intercept	SE	*P*
Asparagus liking	Endometriosis	0.013698	0.023676	0.58392
Asparagus liking	Endometriosis ASRM stages 1,2	0.017212	0.036098	0.650369
Asparagus liking	Endometriosis ASRM stages 3,4	0.018804	0.030919	0.565363
Asparagus liking	Deep endometriosis	− 0.00593	0.049708	0.909002
Asparagus liking	Endometriosis of ovary	0.013981	0.035719	0.709012
Asparagus liking	Endometriosis of pelvic peritoneum	0.016377	0.041883	0.709299
Asparagus liking	Endometriosis of rectovaginal septum and vagina	− 0.01066	0.054708	0.851889
Coffee difference liking	Endometriosis	− 0.0067	0.025059	0.806555
Coffee difference liking	Endometriosis ASRM stages 1,2	− 0.01945	0.032526	0.592044
Coffee difference liking	Endometriosis ASRM stages 3,4	− 0.03577	0.028723	0.30145
Coffee difference liking	Endometriosis of fallopian tube	0.102865	0.166194	0.579774
Coffee difference liking	Unspecified/other endometriosis	− 0.0307	0.056975	0.627409
Coffee difference liking	Endometriosis of pelvic peritoneum	− 0.03736	0.033501	0.346019
Orange juice liking	Endometriosis	− 0.02226	0.055202	0.755965
Orange juice liking	Endometriosis ASRM stages 1,2	− 0.04813	0.052601	0.528206
Orange juice liking	Endometriosis ASRM stages 3,4	− 0.03751	0.043638	0.547941
Orange juice liking	Deep endometriosis	− 0.08621	0.070108	0.434644
Orange juice liking	Unspecified/other endometriosis	− 0.05121	0.083804	0.650802
Orange juice liking	Endometriosis of pelvic peritoneum	− 0.0706	0.068934	0.492396
Orange juice liking	ndometriosis of rectovaginal septum and vagina	− 0.03247	0.077169	0.746428

### Reverse MR analysis

Our findings revealed that the Steiger test P values for the three dietary preference phenotypes, which showed significant causal relationships with EM, as well as various stages and sites of EM, were well below the 0.05 threshold. This highlights the statistical significance of these P-values in confirming the correct directionality of the MR analysis. Additionally, the IVW method used in the reverse MR analyses did not identify any statistically significant reverse causal associations (Table S9, *p* > 0.05).

## Discussion

Our study utilised two-sample MR analysis to investigate the causal relationship between 187 dietary preferences and the risk of 12 types of EM across a cohort of over 500,000 individuals of European ancestry and a total of 64,658 EM cases. This comprehensive analysis, corrected for FDR, revealed significant and consistent causal relationships between three specific dietary preferences and EM, including EM ASRM stages 1–2 and 3–4. Specifically, asparagus liking was found to have a protective effect against EM, whereas different coffee liking and orange juice liking were identified as risk factors. These findings provide a theoretical basis for incorporating dietary habits into the management and treatment strategies for EM.

Additionally, our study revealed causal relationships between mackerel liking, diet-fizzy drink liking, and whiskey liking and EM, excluding EM ASMR stages 1–2 and 3–4. Specifically, preferences for mackerel and fizzy drinks were associated with protective effects against EM. The protective effect of mackerel may be attributed to its high content of omega-3 fatty acids, such as eicosapentaenoic acid (EPA) and docosahexaenoic acid (DHA), which are known for their anti-inflammatory and antioxidant properties^41,42^. These unsaturated fatty acids may inhibit the production of proinflammatory mediators and promote anti-inflammatory pathways, potentially reducing vascular proliferation and tissue fibrosis at EM sites, thereby slowing disease progression^43,44^. Research has shown that mackerel consumption can significantly lower serum cholesterol and triglyceride levels while slightly increasing high-density lipoprotein(HDL) cholesterol levels^45^. Notably, HDL has been identified as a protective factor for EM, whereas elevated triglycerides are a risk factor^46^. Furthermore, studies have shown that supplementation with omega-3 or omega-6 can reduce the serum levels of kisspeptin 1, a key neuropeptide involved in EM^47,48^. Kisspeptin plays a critical role in regulating the hypothalamic‒pituitary‒gonadal axis and may contribute to the pathogenesis of EM by influencing inflammatory responses and oestrogen metabolism. Given the anti-inflammatory properties of omega-3 fatty acids, they may exert beneficial effects on EM by modulating kisspeptin signalling pathways. Therefore, the dietary intake of omega-3-rich foods, such as mackerel, could provide protective effects against EM through anti-inflammatory mechanisms and neuroendocrine regulation. These findings highlight a novel direction for dietary interventions in EM management. Diet fizzy drinks may exert their protective effect through weight management, as they reduce sugar and calorie intake, which is crucial since obesity is considered a risk factor for EM^49^. Conversely, a preference for whiskey and other strong alcoholic beverages is associated with an increased likelihood of EM. Alcohol can potentially disrupt the production of oestrogen, a hormone closely linked to the development of EM^50–52^. Additionally, alcohol consumption may exacerbate the “pain‒stress‒inflammation” cycle, further aggravating EM through proinflammatory and oxidative stress pathways^53,54^. Numerous studies have consistently shown that alcohol intake is associated with an increased likelihood of developing EM^55,56^.

The present study revealed that asparagus preference has a significant protective causal relationship with EM, ASRM stages 1‒2, and ASRM stages 3‒4. The health benefits of asparagus in various other diseases have been widely reported. For example, animal studies have shown that asparagus has positive regulatory effects on the progression of hypertension, type 2 diabetes, liver cancer, and breast cancer^57–60^. Additionally, research has demonstrated that asparagus can effectively increase oestrogen receptor α levels in ovariectomised rats and alleviate polycystic ovary syndrome symptoms through its antioxidant and anti-inflammatory effects via the PRKCA pathway^61,62^. Given that oestrogen and anti-inflammatory/antioxidant factors play crucial roles in the pathogenesis of EM, these findings further support the potential benefits of asparagus in EM. Although there is currently no direct research linking asparagus to EM, this study is the first to reveal the potential importance of asparagus in EM management. Several pathways may be involved in the protective mechanisms of asparagus in EM. First, asparagus is rich in antioxidants and dietary fibre. Antioxidants can neutralize free radicals, reduce oxidative stress, and lower the risk of chronic inflammation^63^. Dietary fibre helps maintain gut health and improve metabolic conditions, providing an additional protective barrier for EM patients^64^. Furthermore, studies have shown that *Asparagus cochinchinensis* root extract can significantly reduce weight gain induced by a high-fat diet and ameliorate lipid abnormalities^65^. Since body weight and lipid levels are important risk factors for EM, these findings further support the potential mechanisms by which asparagus may exert protective effects in EM. We also found that asparagus preference significantly protects against four different types of EM, including deep EM, ovarian EM, pelvic peritoneal EM, and rectovaginal septum and vaginal EM. The mechanisms of these protective effects may vary depending on the specific site. In deep EM, antioxidants and anti-inflammatory compounds in asparagus help reduce oxidative stress and suppress inflammation, thereby alleviating tissue fibrosis^66^. In ovarian EM, folic acid and B vitamins in asparagus support the health of oocytes, whereas antioxidants and potassium improve blood circulation, potentially reducing cyst formation^20,67^. In pelvic peritoneal EM, the anti-inflammatory compounds of asparagus can inhibit the production of inflammatory mediators, and dietary fibre may help modulate the gut microbiota, indirectly reducing inflammation^68–70^. In the rectovaginal septum and vaginal EM, the minerals and vitamin C in asparagus promote collagen synthesis, increase tissue resilience, and protect mucosal integrity, reducing the invasion of adjacent tissues^71,72^. Overall, asparagus exerts protective effects through multiple mechanisms at different anatomical sites of EM, providing scientific evidence for its potential role in EM management and offering valuable insights for dietary intervention strategies.

Current research indicates that a preference for orange juice is associated with an increased likelihood of EM. An RCT has shown that consuming orange juice for two months can promote weight loss, reduce energy and nutrient intake, improve lipid profiles, and enhance insulin sensitivity^73^. However, this study included vitamin D3 (2000 IU) in orange juice, and it did not significantly impact triglycerides or HDL, both of which are linked to EM^46^. Although orange juice is rich in vitamin C and antioxidants, its high fructose content may lead to metabolic imbalances, increasing the risk of insulin resistance. Insulin resistance is closely associated with chronic inflammation, a critical mechanism in the pathogenesis of EM^74,75^. Additionally, excessive fructose intake may disrupt lipid metabolism, leading to hormonal imbalances and exacerbating EM symptoms^46^. A preference for orange juice has been linked to an increased likelihood of various EM subtypes, including deep EM, unspecified/other EM, pelvic peritoneal EM, and rectovaginal septum and vaginal EM. In deep EM, the high sugar content in orange juice may exacerbate aerobic glycolysis, which could facilitate the proliferation of ectopic cells and worsen their condition^76^. In unspecified/other EMs, excessive sugar intake can increase systemic inflammation levels, stimulating the growth of ectopic cells^75^. For pelvic peritoneal EM, sugars in orange juice may intensify inflammatory responses, which could alter the peritoneal environment, facilitating cell infiltration and fibrosis progression and thereby increasing risk^77^. In rectovaginal septum and vaginal EM, the acidic components in orange juice may weaken the mucosal barrier, making ectopic lesions more likely to invade and spread to adjacent tissues^78^.

Research on the relationship between coffee consumption and EM has yielded mixed results. A previous study suggested that coffee had no significant impact on EM^79^, whereas other studies have indicated that high caffeine intake (> 300 mg/day) may be associated with an increased likelihood of EM^80^. The present study is the first to demonstrate a significant causal relationship between different coffee preferences and increased EM risk. Interestingly, different coffee preferences have varying impacts on EM. Specifically, a preference for sugar-sweetened coffee is linked to a greater risk of intestinal EM (IVW method, *P* = 0.049, OR = 2.684, 95% CI: 1.004–7.178), possibly due to the increased insulin resistance and chronic inflammation caused by high sugar intake, which are closely related to EM progression. Excessive sugar consumption may also lead to fluctuations in oestrogen levels, a key factor in EM development^81^. Conversely, a preference for unsweetened coffee was found to have a protective effect against deep EM (IVW method, *P* = 0.012, OR = 0.623, 95% CI: 0.432–0.899). The caffeine and polyphenols in unsweetened coffee are believed to possess antioxidant and anti-inflammatory properties^82^, which can alleviate EM symptoms and inhibit disease progression. Notably, caffeine intake is associated with fluctuations in oestrogen levels, and oestrogen is a critical factor in EM progression^83^. These compounds may exert their beneficial effects by influencing oestrogen metabolism, reducing oxidative stress, and decreasing inflammation. Additionally, coffee preference was associated with an increased likelihood of EM in the fallopian tube, unspecified/other EM, and pelvic peritoneal EM. The mechanisms underlying these associations likely involve caffeine-induced fluctuations in oestrogen levels, exacerbation of local inflammation, and metabolic imbalances, all of which can contribute to the growth and spread of ectopic cells^75,84,85^. These findings highlight the complex role of coffee in EM development and underscore the importance of considering dietary habits in EM management and treatment.

In conclusion, this study highlights the complex roles of specific dietary preferences in the pathophysiology of EM, emphasising the potential of dietary interventions in the prevention and treatment of EM, which could have significant implications for clinical management. These findings suggest that health care providers may improve patient outcomes by considering dietary adjustments in EM management strategies, reducing reliance on surgical treatments and alleviating medical and social burdens^86^. Increasing asparagus intake while reducing the consumption of coffee and orange juice could help slow disease progression. Additionally, evaluating dietary preferences could offer new insights for early screening and staging of EM, contributing to more comprehensive treatment plans. Future research should explore the precise mechanisms through which asparagus impacts EM, potentially leading to the development of effective therapeutic agents and intervention targets.

Despite providing new insights into the relationship between dietary preferences and EM, our study has several limitations. First, the study is based on data from a European population, which may limit the generalizability of the findings to other ethnicities or populations. Additionally, the sample size and the reliance on self-reported dietary preferences could introduce bias. Moreover, while MR analysis is a powerful tool for establishing causal relationships, the results still require experimental validation to confirm the underlying mechanisms involved. Future research should consider conducting multicentre studies, including diverse populations and ethnic groups, with long-term follow-up to further validate and expand upon these findings.

## Conclusion

This study revealed that preferences for asparagus significantly reduce the risk of developing EM, whereas preferences for different types of coffee and orange juice increase this risk. These findings highlight the importance of dietary factors in managing EM. Future approaches may include adjusting dietary patterns as a potential strategy for managing this condition, warranting further research to explore these relationships and their therapeutic implications.

## Electronic supplementary material

Below is the link to the electronic supplementary material.

Supplementary Material 1

## Data Availability

The GWAS summary statistics for EM are available in the Finnish database version R.11 (https://r11.finngen.fi/). The GWAS summary statistics for dietary preferences were derived from a previous study. The code and data used in the present study can be obtained from the corresponding author upon reasonable request.

## Abbreviations

EM: Endometriosis
RCT: Randomised controlled trial
PCOS: Polycystic Ovary Syndrome
MR: Mendelian randomization
IVs: Instrumental variables
LD: Linkage disequilibrium
SNPs: Single nucleotide polymorphisms
GWAS: Genome-Wide Association Study
IVW: Inverse-variance weighted
OR: Odds ratio
CI: Confidence interval
ASRM: American Society for Reproductive Medicine
FDR: False discovery rate
MR-PRESSO: Mendelian Randomization Pleiotropy RESidual Sum and Outlier
EPA: Eicosapentaenoic acid
DHA: Docosahexaenoic acid
HFD: High-fat diet
HDL: High-density lipoprotein
